# Vol.15 (2005), Supplement_II pp.S120

**DOI:** 10.2188/jea.15.197_2

**Published:** 2005-09-27

Wrong:Received: December 24, 2005

Right:Received: December 25, 2004

